# A Penalized Regression Method for Genomic Prediction Reduces Mismatch between Training and Testing Sets

**DOI:** 10.3390/genes15080969

**Published:** 2024-07-23

**Authors:** Osval A. Montesinos-López, Cristian Daniel Pulido-Carrillo, Abelardo Montesinos-López, Jesús Antonio Larios Trejo, José Cricelio Montesinos-López, Afolabi Agbona, José Crossa

**Affiliations:** 1 Facultad de Telemática, Universidad de Colima, Colima 28040, Mexico; osval78t@gmail.com (O.A.M.-L.); c.pulido@gmail.com (C.D.P.-C.); 2 Centro Universitario de Ciencias Exactas e Ingenierías (CUCEI), Universidad de Guadalajara, Guadalajara 44430, Mexico; 3 Facultad de Ciencias de la Educación, Universidad de Colima, Colima 28040, Mexico; jesus_larios@ucol.mx; 4 Department of Public Health Sciences, University of California Davis, Davis, CA 95616, USA; joscriml@gmail.com; 5 International Institute of Tropical Agriculture (IITA), Ibadan 200113, Nigeria; aabgona@gmail.com; 6 Molecular & Environmental Plant Sciences, Texas A&M University, College Station, TX 77843, USA; 7 International Maize and Wheat Improvement Center (CIMMYT), Km 45, Carretera Mexico-Veracruz, Texcoco 52640, Mexico; 8 Louisiana State University, Baton Rouge, LA 70803, USA; 9 Distinguished Scientist Fellowship Program and Department of Statistics and Operations Research, King Saud University, Riyah 11451, Saudi Arabia; 10 Colegio de Postgraduados, Montecillos 56230, Mexico

**Keywords:** genomic selection, mismatch, Ridge regression, Lasso regression, Elastic Net regression, weighted regression

## Abstract

Genomic selection (GS) is changing plant breeding by significantly reducing the resources needed for phenotyping. However, its accuracy can be compromised by mismatches between training and testing sets, which impact efficiency when the predictive model does not adequately reflect the genetic and environmental conditions of the target population. To address this challenge, this study introduces a straightforward method using binary-Lasso regression to estimate *β* coefficients. In this approach, the response variable assigns 1 to testing set inputs and 0 to training set inputs. Subsequently, Lasso, Ridge, and Elastic Net regression models use the inverse of these *β* coefficients (in absolute values) as weights during training (WLasso, WRidge, and WElastic Net). This weighting method gives less importance to features that discriminate more between training and testing sets. The effectiveness of this method is evaluated across six datasets, demonstrating consistent improvements in terms of the normalized root mean square error. Importantly, the model’s implementation is facilitated using the glmnet library, which supports straightforward integration for weighting *β* coefficients.

## 1. Introduction

Genomic selection (GS) is a transformative methodology in plant breeding, offering substantial reductions in phenotyping costs. GS achieves this by using a statistical machine learning algorithm trained on a reference population that includes both phenotypic and genotypic data. The trained model then predicts phenotypic values for a target population based solely on their genotypic data [1]. Consequently, GS is widely adopted in breeding programs, enabling the early selection of candidate lines without the necessity of phenotyping.

GS has been successfully implemented in various crop breeding programs, leading to significant advancements [2]. Notable examples include maize, where GS has enhanced yield, disease resistance, and drought tolerance. In wheat, GS has improved grain yield, disease resistance, and quality traits [3,4]. For rice, GS has increased yield, pest and disease resistance, and environmental adaptability [5,6]. In soybean, GS has boosted yield, disease resistance, and oil/protein content [7,8]. Barley has seen enhanced yield, malting quality, and disease resistance due to GS [9,10]. In cassava, GS has improved yield, disease resistance, and nutritional content [11,12]. Potato breeding has benefited from increased yield, disease resistance, and tuber quality through GS [13,14]. Lastly, GS has improved yield, disease resistance, and fruit quality in tomato breeding. These examples underscore the effectiveness of GS in enhancing key traits across a diverse range of crops.

According to Montesinos-López et al. [1], the successful implementation of GS in crop breeding depends on several critical factors. High-quality and extensive genotypic and phenotypic data are essential for training accurate predictive models. A large, diverse reference population that accurately represents the genetic diversity of the target population enhances model robustness. The choice of advanced statistical models and machine learning algorithms, along with adequate computational resources, also significantly impacts the predictive power of GS. Traits with higher heritability and those influenced by fewer genes are typically predicted more accurately. Environmental factors and genotype-by-environment interactions must be considered to improve prediction accuracy for traits affected by external conditions. Clear breeding objectives, well-designed programs, and cost considerations are crucial for the effective integration of GS. Additionally, the presence of skilled personnel with expertise in quantitative genetics, statistics, bioinformatics, and computational biology is vital. Collaborative efforts among breeding programs, research institutions, and industry stakeholders can provide the necessary resources, data, and expertise, further enhancing the successful application of GS in crop breeding.

A significant mismatch between the reference (training) population and the target (testing) population can significantly undermine the accuracy of GS predictions [15]. This issue arises when the genetic composition, allele frequencies, and linkage disequilibrium patterns differ between the two populations. Such discrepancies can lead to incorrect marker–trait associations and biased predictions. Additionally, genotype-by-environment interactions may not be adequately captured if the populations experience different environmental conditions. Variability in phenotypic expression and genetic backgrounds further complicates prediction accuracy. To mitigate these problems, it is essential to ensure that the reference population is representative of the target population, incorporating diverse genetic backgrounds and environmental conditions. This approach enhances the reliability of GS predictions by better capturing the genetic and environmental complexities present in the target population.

In this context, a method using penalized Lasso, Ridge, and Elastic Net regression [16] is proposed to reduce the mismatch between training and testing sets. This proposed method is evaluated in eight real datasets, each containing multiple environments. The model is implemented in a uni-environment fashion, with each environment containing several families. Given that the genetic material of each family differs, the training set in each dataset consists of all families minus one, and the remaining family is used as the testing set. We compare the proposed method where the coefficient of regression is weighted by the absolute value of the regression coefficient with conventional penalized methods, assessing prediction performance in terms of normalized root mean square errors.

## 2. Material and Methods

### 2.1. Datasets

Table 1 has details of the 8 datasets included in this study. The data used were obtained from Montesinos-Lopez et al. (2023) [17] and comprise two crops, Maize and Soybean, which have different numbers of DNA sequences and different numbers of environments (location–year combination) and numbers of traits.

### 2.2. Statistical Model

In a general context, we have a covariate vector $X={\left(X_{1},\;\ldots ,\;X_{p}\right)}^{T}$, such as molecular markers, and we want to use this information to predict or explain how this variable affects a real-value response Y (of size *n*), for example, grain yield or any other quantitative trait the breeders want to predict. The linear multiple regression model assumes a relationship given by

(1) $$Y=\beta_{0}+\sum_{j=1}^{p}X_{j}\beta_{j}+\epsilon$$

where ϵ is a random error vector with mean **0**, $E\left(\epsilon \right)=0$ and is independent of X. This error is included in the model to capture measurement errors and the effects of other unregistered explanatory variables that can help explain the mean response. Then, the conditional mean of this model is $E\left(Y|X\right)=\beta_{0}+\sum_{j=1}^{p}X_{j}\beta_{j}$, and the conditional distribution of Y given X is only affected by the information of X.

To estimate the parameters $\beta_{0}$ and $\beta ={\left(\beta_{1},\;\ldots ,\;\beta_{p}\right)}^{T}$, we usually have a set of data $\left(x_{i}^{T},y_{i}\right)$, i=1, …, n, often known as training data, where $x_{i}={\left(x_{i0},\;x_{i1},\;\ldots ,\;x_{ip}\right)}^{T}$ is a vector of feature measurement and $y_{i}$ is the response variable measurement corresponding to the i-th individual drawn. In the context of large p and small n, the ordinary least squares method that does not penalized the parameters is used, and in this context, the estimates are not optimal. One common method to estimate β and $\beta_{0}$ when *p* >> *n* is the Elastic regression (ENet) method (1), which consists of taking the ($\beta_{0},\beta )$ value that minimizes the penalized residual sum of squares [1,16], defined as

(2) $$PRSS_{\lambda }\left(\beta \right)=\sum_{i=1}^{n}{\left(y_{i}-\beta_{0}-\sum_{j=1}^{p}x_{ij}\beta_{j}\right)}^{2}+\lambda \left((1-\alpha )\sum_{j=1}^{p}\beta_{j}^{2}+\alpha \sum_{j=1}^{p}\left|\beta_{j}\right|\right)\;$$

where λ≥0 is the regularization parameter, which determines the level or degree to which the *β* coefficients are shrunk towards zero, α∈(0,1). When λ=0 and α = 0, the ordinal least square (OLS) is the solution to the *β* coefficients, but when λ is large, $PRSS_{\lambda }\left(\beta \right)$ is dominated by the penalization term, and the OLS solution must shrink towards 0 [18]. In general, when the number of parameters (*p*) to be estimated is larger than the number of observations (*n*), the estimator can be highly variable. In this situation, the intuition of penalized regression tries to alleviate this by constraining the *β* coefficients [19]. Also, it is important that when α = 0, the loss function given in (1) is reduced to the loss function of Ridge regression (Ridge; [1,16]) while when α = 1, the loss function corresponds to the Lasso regression [16].

### 2.3. Proposed Approach

The proposed method is described below in 4 steps.

- **Step 1**. We assume that we have a uni-environment or single-environment dataset in which there are at least two families, and for each family, there are some lines. Since our goal is to predict a complete family, the information of this family constitutes the testing set {X_tst, y_tst}, and the information of the remaining families is the training set {X_trn, y_trn}. Then, in the original dataset {X_trn, y_trn, X_tst, y_tst}, we remove the original response variable column {y_trn, y_tst} and add a fictitious (new) response variable column {yf_trn, yf_tst} that replaces the source of the data with zero (that is, yf_trn = 0) for samples (observations) on the training set and with one (i.e., yf_tst = 1) for the samples in the testing set [17]. In other words, the fictitious response variables with 0 s correspond to the remaining families, and those with 1 s correspond to the family we want to predict. Note that since the response variable is a fictitious response variable, with a training label (yf_trn = 0) and a testing label (yf_tst = 1), not the original response variable, we should avoid any complication in defining these two populations when performing the random cross-validation and also avoid any type of data leakage confusion.
- **Step 2**. We then implement the Lasso regression with inputs {X_trn, yf_trn, X_tst, yf_tst} and we extract the *β* coefficients ${(\beta }_{bin}={\left(\beta_{1,bin},\;\ldots ,\;\beta_{p,bin}\right)}^{T})$ for each marker.
- **Step 3**. The next step is to compute weights as $w_{j}$ = 1/abs($\beta_{j,\;bin}$), where j=1, …, p, p denotes the number of markers.
- **Step 4**. Then, the weights computed in step 3 are used to train a weighted Lasso and Ridge regression. Regarding the Weighted Lasso (WLasso), the loss function used to train the models is given as follows:

(3) $$PRSS_{\lambda }\left(\beta_{w_{L}}\right)=\sum_{i=1}^{n}{\left(y_{i}-\beta_{0}-\sum_{j=1}^{p}x_{ij}\beta_{j}\right)}^{2}+\lambda \sum_{j=1}^{p}w_{j}\left|\beta_{j}\right|$$

Meanwhile, for the weighted Ridge regression (WRidge), the weighted loss function is equal to

(4) $$PRSS_{\lambda }\left(\beta_{R}\right)=\sum_{i=1}^{n}{\left(y_{i}-\beta_{0}-\sum_{j=1}^{p}x_{ij}\beta_{j}\right)}^{2}+\lambda \sum_{j=1}^{p}w_{j}\beta_{j}^{2}$$

and for the weighted Elastic net regression (WENet), the weighted loss function is equal to

(5) $$PRSS_{\lambda }\left(\beta_{R}\right)=\sum_{i=1}^{n}{\left(y_{i}-\beta_{0}-\sum_{j=1}^{p}x_{ij}\beta_{j}\right)}^{2}+\lambda \left(\left(1-\alpha \right)\sum_{j=1}^{p}w_{j}\beta_{j}^{2}+\alpha \sum_{j=1}^{p}w_{j}\left|\beta_{j}\right|\right)$$

To implement these loss functions, we used the glmnet library in [16], which is implemented in R statistical software (v. 2024) [20].

### 2.4. How to Avoid Data Leakage

Avoiding data leakage in genomic prediction studies is crucial to ensure the validity and generalizability of your model’s performance. Data leakage occurs when information from outside the training dataset is used to create the model, leading to overly optimistic performance estimates. This can be avoided by properly separating training sets from testing sets; this means that no data points used for training the model should be included in the testing set, and vice versa.

Furthermore, we use cross-validation techniques where the dataset is split into *k* subsets, and the model is trained on *k* − 1 subsets and tested on the remaining subset. This helps in validating the model’s performance across different subsets of the data, minimizing the risk of data leakage. In the prediction of longitudinal data, it is important to ensure that the training data precede the testing data chronologically. This simulates real-world scenarios where predictions are made on future, unseen data.

We must ensure that there are no duplicates or near-duplicates between the training and testing sets. In genomic studies, this means carefully managing how genotypes and phenotypes are split, ensuring no overlap. Feature selection should be performed within the cross-validation loop. Performing feature selection before splitting the data can lead to information from the test set leaking into the model, thereby inflating performance metrics. We should use resampling techniques like bootstrapping within the training data only. We must not mix data points from the test set during resampling, as this can cause leakage.

### 2.5. Evaluation of Prediction Accuracy

Our evaluation procedure preserves data leakage by employing a cross-validation approach wherein each iteration excludes one family at a time from the training set, and the entire proposed approach described above is applied. Therefore, data from the testing set do not influence the weightings derived from the training set.

Specifically, in each iteration, data from a single family served as the testing set, while data from the remaining families constituted the training set [1]. The number of iterations corresponded to the total number of families, ensuring that each family was used as the testing set exactly once. This method was implemented to evaluate the model’s capability to predict information for an entire family based on data from other diverse families. To compare the predictive performance of the proposed method (WRidge, WLasso, and WENet) with the conventional glmnet method (Ridge, Lasso and ENet), the normalized root mean square error (NRMSE) was employed as the evaluation metric.

The NRMSE, a measure of prediction error, was calculated by first computing the square root of the mean square error (MSE) and then dividing this value by the average of the observed values in the testing set. The NRMSE is a metric used to evaluate the accuracy of a predictive model, particularly useful when comparing models with different scales. It is a normalized version of the root mean square error (RMSE), which adjusts for the scale of the data, making it easier to interpret and compare across different datasets. NRMSE = 0: perfect predictions. NRMSE > 0: the further the value from 0, the worse the model’s predictions. NRMSE < 1: indicates a good fit, depending on the context and domain. Some advantages of NRMSE are that it is scale-invariant as it adjusts to the scale of the data, making it easier to compare across different datasets, and has an intuitive interpretation because it provides a relative measure of the prediction error in relation to the range of the observed values. Using NRMSE helps provide a more standardized assessment of model performance, making it particularly useful in genomic prediction studies where traits can have varying scales.

## 3. Results

**Figure 1:**
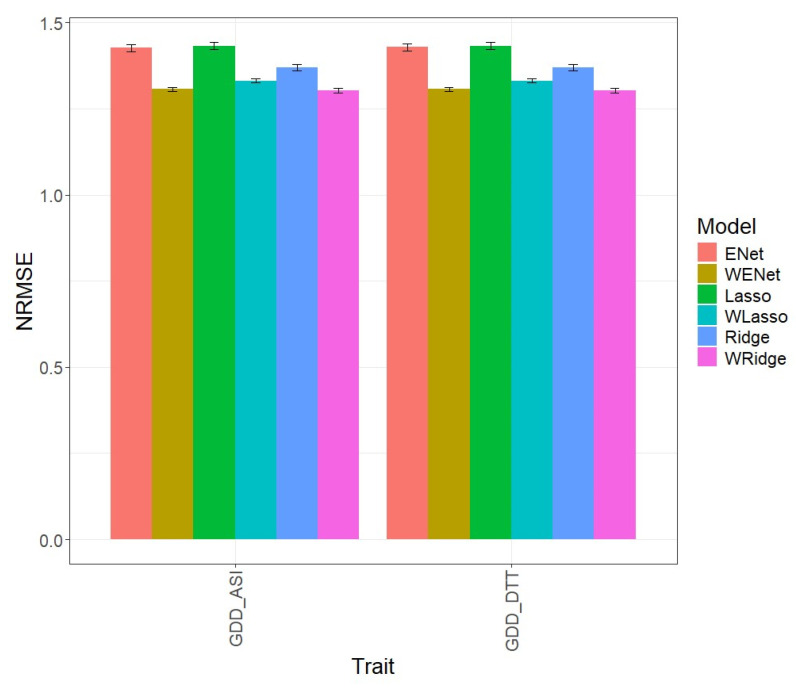
Normalized root mean square error (NRMSE) for the “Maize_1” dataset. A comparison is presented for the 6 evaluated models (Enet, Lasso, Ridge, WEnet, WLasso, and WRidge) and the 2 traits (GDD_ASI and GDD_DTT).

**Figure 2:**
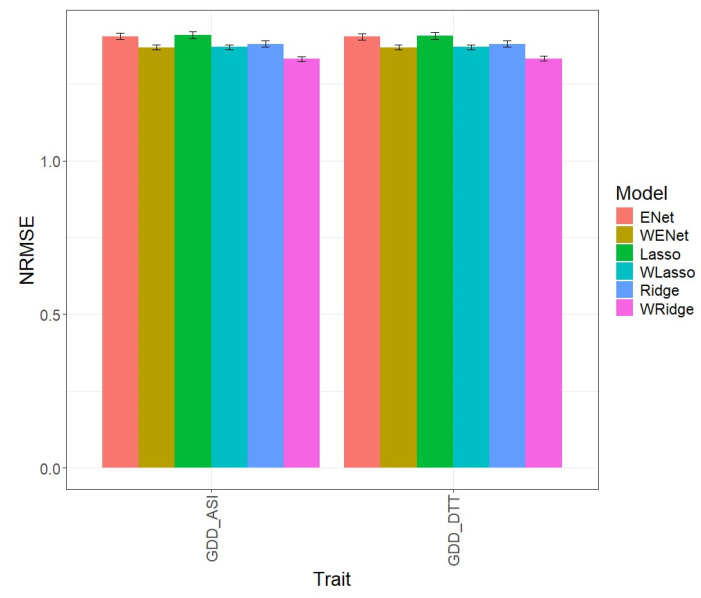
Normalized root mean square error (NRMSE) for the “Maize 3” dataset. A comparison is presented for the 6 evaluated models (Enet, Lasso, Ridge, WEnet, WLasso, and WRidge) and the 2 traits (GDD_ASI and GDD_DTT).

**Figure 3:**
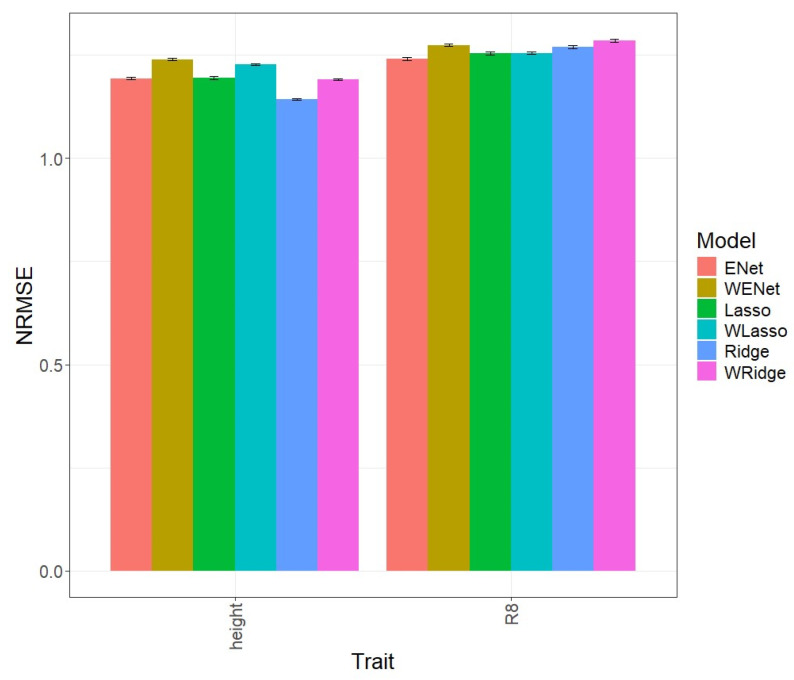
Normalized root mean square error (NRMSE) for the “Soybean 1” dataset. A comparison is presented for the 6 evaluated models (Enet, Lasso, Ridge, WEnet, WLasso, and WRidge) and the 2 traits (Height and R8).

**Figure 4:**
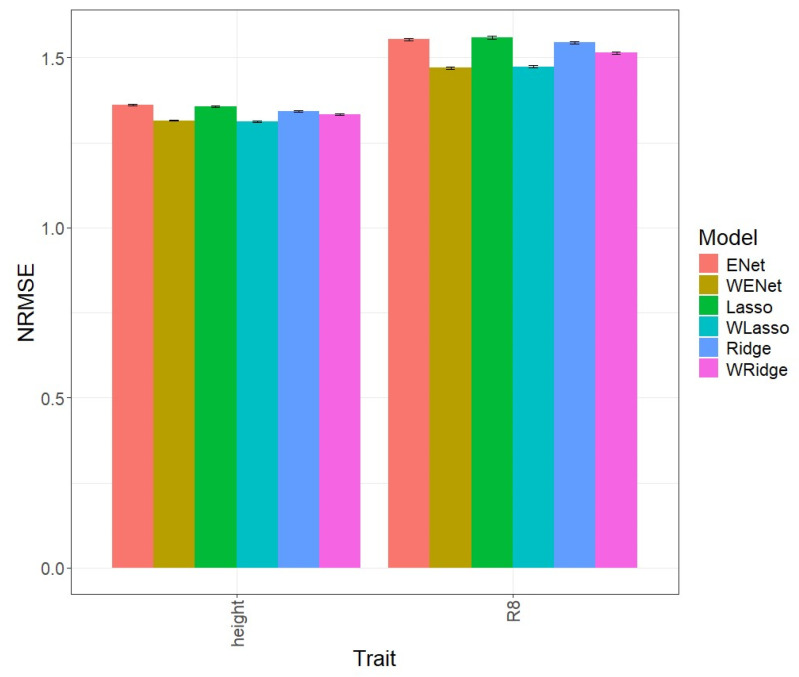
Normalized root mean square error (NRMSE) for the “Soybean 2” dataset. A comparison is presented for the 6 evaluated models (Enet, Lasso, Ridge, WEnet, WLasso, and WRidge) and the 2 traits (Height and R8).

**Figure 5:**
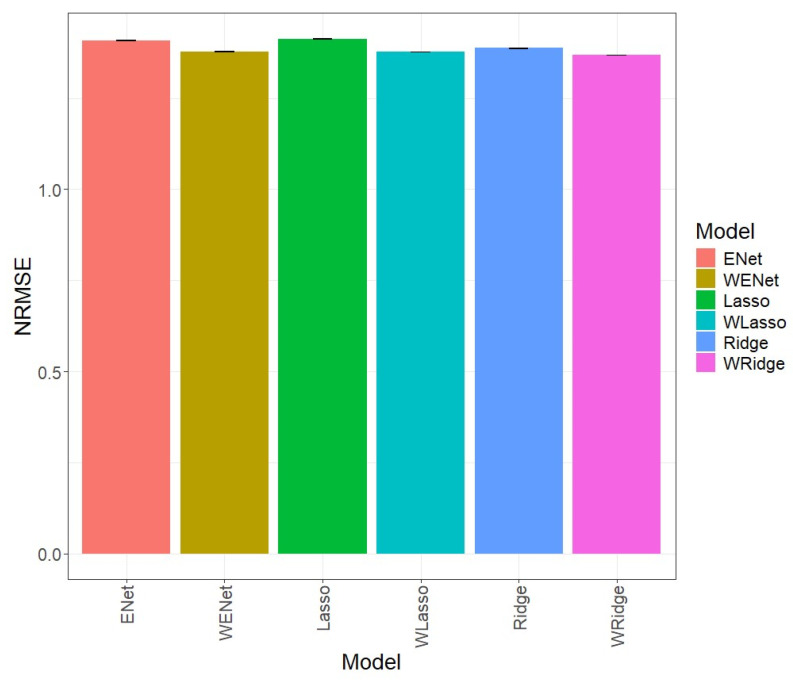
Normalized root mean square error (NRMSE) for all datasets. A comparison is presented for the 6 evaluated models (Enet, Lasso, Ridge, WEnet, WLasso, and WRidge).

**Figure A1:**
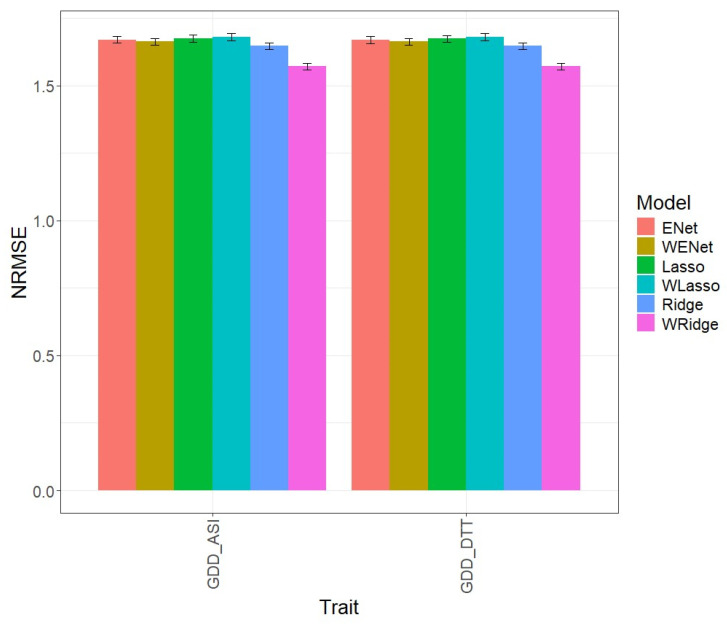
Dataset Maize 2. Normalized root mean square error (NRMSE) for the Maize_2 dataset. A comparison is presented for the 6 evaluated models (Enet, Lasso, Ridge, WEnet, WLasso, and WRidge) and the 2 traits (GDD_ASI and GDD_DTT).

**Figure A2:**
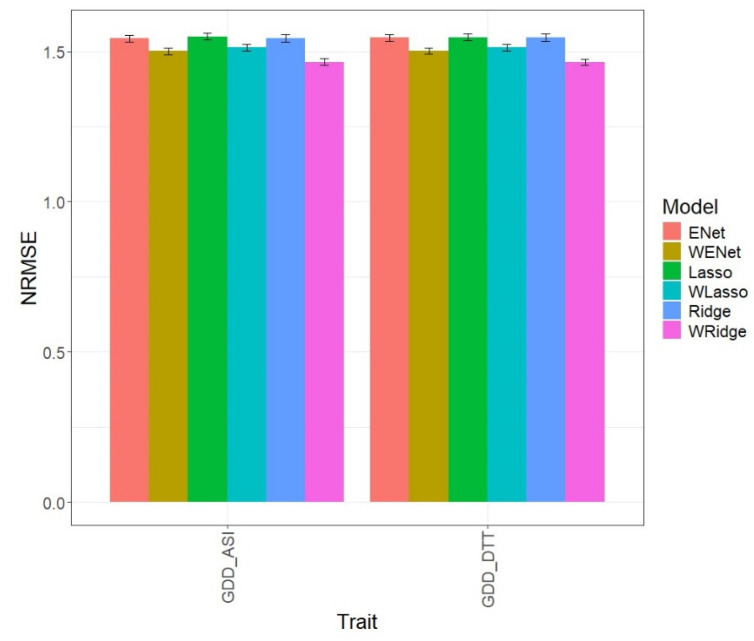
Dataset Maize 4. Normalized root mean square error (NRMSE) for the Maize_4 dataset. A comparison is presented for the 6 evaluated models (Enet, Lasso, Ridge, WEnet, WLasso, and WRidge) and the 2 traits (GDD_ASI and GDD_DTT).

**Figure A3:**
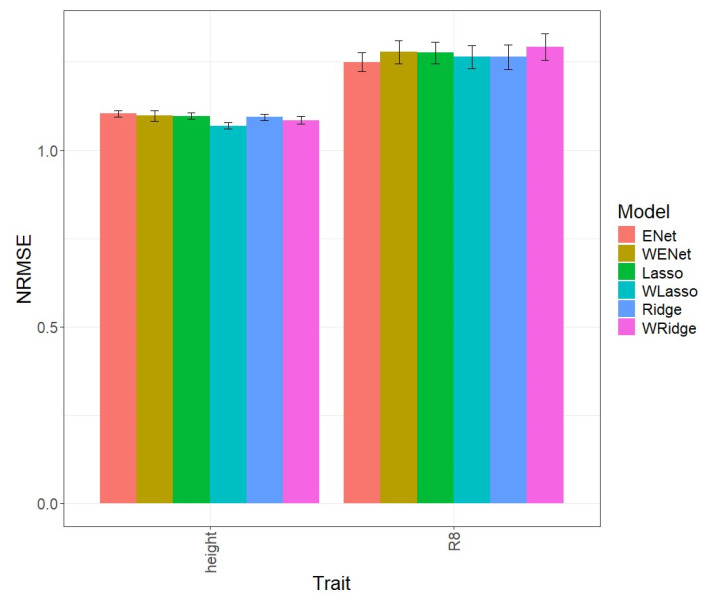
Dataset Soybean 3. Normalized root mean square error (NRMSE) for the Soybean_3 dataset. A comparison is presented for the 6 evaluated models (Enet, Lasso, Ridge, WEnet, WLasso, and WRidge) and the 2 traits (Height and R8).

**Figure A4:**
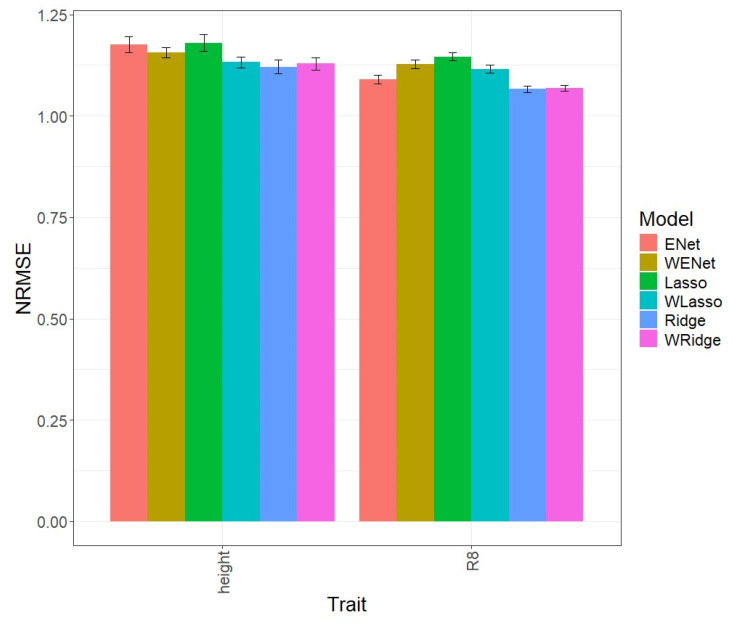
Dataset Soybean 4. Normalized root mean square error (NRMSE) for the Soybean_4 dataset. A comparison is presented for the 6 evaluated models (Enet, Lasso, Ridge, WEnet, WLasso, and WRidge) and the 2 traits (Height and R8).

The results are provided in five sections that contain the results of the datasets Maize_1, Maize_3, Soybean_1, and Soybean_2 and across all of these datasets, presented in Figure 1, Figure 2, Figure 3, Figure 4 and Figure 5 and Table 2 and Table 3. The results for the rest of the datasets (Maize_2, Maize_4, Soybean_3, Soybean_4) are given in Appendix A containing Table A1 and Figure A1, Figure A2, Figure A3 and Figure A4.

### 3.1. Maize_1

Figure 1 shows the prediction performance in terms of the normalized root mean square error (NRMSE) for Maize Dataset 1. The first three original prediction models (ENet, Lasso, Ridge) were compared with their weighted versions (WENet, WLasso, WRidge) using two traits for these predictions: GDD_ASI and GDD_DTT.

In the first comparison, we can observe that, in terms of NRMSE for the GDD_ASI trait, the ENet model, with an NRMSE value of 1.427, was inferior to the weighted WENet model, with an NRMSE value of 1.307, being superior in prediction by 9.185%. Similarly, for the GDD_DTT trait, the WENet model, with an NRMSE value of 1.307, also outperformed the Enet, with an NRMSE value of 1.429, with a difference of 9.358%. The individual values can be observed in more detail in Table 2.

In the second comparison, we can observe that, in terms of NRMSE for the GDD_ASI trait, the Lasso model, with an NRMSE value of 1.433, was inferior to the weighted WLasso model, with an NRMSE value of 1.332, surpassing it in prediction by 7.567%. Similarly, for the GDD_DTT trait, the WLasso model, with an NRMSE value of 1.332, also outperformed the Lasso, with an NRMSE value of 1.433, with a difference of 7.564%. The individual values can be observed in more detail in Table 2.

In the third comparison, we can observe that, in terms of NRMSE for the GDD_ASI trait, the Ridge model, with an NRMSE value of 1.371, was inferior to the weighted WRidge model, with an NRMSE value of 1.303, being superior in prediction by 5.171%. Similarly, for the GDD_DTT trait, the WRidge model, with an NRMSE value of 1.303, also outperformed the Ridge, with an NRMSE value of 1.371, with a difference of 5.180%. The individual values can be observed in more detail in Table 2.

### 3.2. Maize_3

In the first comparison (Figure 2), we can observe that, in terms of NRMSE for the GDD_ASI trait, the ENet model, with an NRMSE value of 1.405, was inferior to the WENet model, with an NRMSE value of 1.369, which outperformed it in prediction by 2.647%. Similarly, for the GDD_DTT trait, the WENet model, with an NRMSE value of 1.369, was also superior to Enet, with an NRMSE value of 1.404, with an advantage of 2.549%. The individual values can be observed in more detail in Table 2.

In the second comparison (Figure 2), we can observe that, in terms of NRMSE for the GDD_ASI trait, the Lasso model, with an NRMSE value of 1.409, was inferior to the WLasso model, with an NRMSE value of 1.370, which outperformed it in prediction by 2.866%. Similarly, for the GDD_DTT trait, the WLasso model, with an NRMSE value of 1.370, was also superior to Lasso, with an NRMSE value of 1.407, with an advantage of 2.721% (see Table 2).

For the third comparison displayed in Figure 2, note that regarding the NRMSE for the GDD_ASI trait, the Ridge model, with an NRMSE value of 1.380, was inferior to the WRidge model, with an NRMSE value of 1.331, which outperformed it in prediction by 3.656%. Similarly, for the GDD_DTT trait, the WRidge model, with an NRMSE value of 1.333, was also superior to Ridge, with an NRMSE value of 1.381, with an advantage of 3.598%. The individual values can be observed in more detail in Table 2.

### 3.3. Soybean_1

In the first comparison (Figure 3), it is evident that, in terms of NRMSE for the R8 trait, the ENet model, with an NRMSE value of 1.241, outperformed the weighted WENet model, with an NRMSE value of 1.274. The weighted model, in this instance, fell short in prediction by 2.621%. Similarly, for the Height trait, the WENet model, with an NRMSE value of 1.239, was inferior to Enet, with an NRMSE value of 1.193, exhibiting a shortfall of 3.729%. Detailed individual values can be observed in Table 2.

In Figure 3, it is noticeable that, in terms of NRMSE for the R8 trait, the Lasso model, with an NRMSE value of 1.254, surpassed the weighted WLasso model, with an NRMSE value of 1.255, by 0.04%. Likewise, the weighted WLasso model, with an NRMSE value of 1.227, fell short in prediction for the Height trait, being inferior to the Lasso model, with an NRMSE value of 1.195, by 2.675%. Detailed individual values can be observed in Table 2.

Figure 3 displays that in terms of NRMSE for the R8 trait, the Ridge model, with an NRMSE value of 1.270, outperformed the weighted WRidge model, with an NRMSE value of 1.284. The weighted model, in this instance, fell short in prediction by 1.112%. Similarly, for the Height trait, the WRidge model, with an NRMSE value of 1.191, was inferior to Ridge, with an NRMSE value of 1.143, exhibiting a shortfall of 4.018%. Detailed individual values can be observed in Table 2.

### 3.4. Soybean_2

In the first comparison (Figure 4), it is evident that, in terms of NRMSE for the R8 trait, the ENet model, with an NRMSE value of 1.554, was inferior to the weighted WENet model, with an NRMSE value of 1.470, which outperformed it in prediction by 5.711%. Similarly, for the Height trait, the WENet model, with an NRMSE value of 1.316, also surpassed Enet, with an NRMSE value of 1.361, exhibiting superiority of 3.427%. Detailed individual values can be observed in Table 2.

In Figure 4, it is noticeable that, in terms of NRMSE for the R8 trait, the Lasso model, with an NRMSE value of 1.559, was inferior to the weighted WLasso model, with an NRMSE value of 1.473, which outperformed it in prediction by 5.828%. Similarly, for the Height trait, the WLasso model, with an NRMSE value of 1.313, also surpassed Lasso, with an NRMSE value of 1.357, exhibiting superiority of 3.317%. Detailed individual values can be observed in Table 2.

Figure 4 displays that regarding NRMSE for the R8 trait, the Ridge model, with an NRMSE value of 1.545, was inferior to the weighted WRidge model, with an NRMSE value of 1.515, which outperformed it in prediction by 2.013%. Similarly, for the Height trait, the WRidge model, with an NRMSE value of 1.332, also surpassed Ridge, with an NRMSE value of 1.342, exhibiting superiority of 0.682%. Detailed individual values can be observed in Table 2.

### 3.5. Across_Data

In the first comparison (Figure 5), it is evident that, in terms of NRMSE, the ENet model, with an NRMSE value of 1.409, was inferior to the weighted WENet model, with an NRMSE value of 1.378, which outperformed it in prediction by 2.196%. Detailed individual values can be observed in Table 3. Figure 5 shows that the Lasso model, with an NRMSE value of 1.413, was worse regarding the weighted WLasso model, with an NRMSE value of 1.377, which outperformed it in prediction by 2.630%. Detailed individual values can be observed in Table 3. On the other hand, the Ridge model, with an NRMSE value of 1.387, was superior to the weighted WRidge model, with an NRMSE value of 1.369, which outperformed it in prediction by 1.309%.

## 4. Discussion

There is no doubt that climate change and a growing global population are increasing the pressure to boost productivity worldwide [21]. Therefore, innovative methods like GS are very appealing, as they have the potential to significantly enhance crop productivity on a global scale. For this reason, the adoption of GS is being accelerated in many public and private breeding programs due to its tremendous potential. However, like many new technologies, the practical and successful implementation of GS depends on several factors that impact its accuracy. Consequently, research is being conducted to mitigate these factors affecting its performance. In this study, we propose a method to increase the efficiency of GS in contexts where there is a limited relationship between the training and testing sets.

The core idea of the proposed method is to assign greater weight to features (markers) that less distinctly differentiate between the training and testing sets and, conversely, to assign less weight to those that do. To achieve this, the method first trains a binary classifier (Lasso regression) with a fictitious response variable that assigns a value of 1 to testing set inputs and 0 to training set inputs. From this first step, the *β* coefficients are obtained and used to compute weights for the second step, which uses the response variable of interest. These weights are calculated as the inverse of the absolute values of the *β* coefficients from the first stage.

The proposed method demonstrated superior performance across various datasets. Specifically, WENet outperformed Enet by 2.196% in terms of NRMSE. Similarly, WLasso exceeded Lasso by 2.63% in terms of NRMSE. Lastly, WRidge surpassed Ridge with a 1.309% improvement in NRMSE. We acknowledge that the margin of improvement is modest; however, the proposed method provides empirical evidence of its ability to enhance prediction accuracy in contexts with significant mismatches between training and testing sets. Therefore, we encourage further exploration and refinement of this method to boost its predictive power. Since in each environment of all datasets, we predict a complete family using the remaining families, we cannot expect good prediction performance when the family to be predicted is genetically distant from those in the training set. Additionally, since families are often genetically distinct from each other, achieving good prediction performance for new families is challenging. Furthermore, predicting a complete family is complicated because markers do not capture all the necessary information for optimal predictions.

Finally, we emphasize that GS is revolutionizing plant and animal breeding by enabling the selection of individuals without the need to measure their phenotypic values. However, its successful implementation faces numerous challenges that must be addressed. This paper underscores the critical importance of understanding that when training and testing materials have significant genetic distance, the predictive quality of GS methodologies deteriorates significantly. Therefore, investigating statistical approaches to mitigate this issue is crucial for enhancing the robustness of GS methodologies.

### 4.1. Why Weighting Markers Might Be Useful for Enhancing Genomic Prediction

The usefulness of weighting markers in genomic prediction can vary depending on the characteristics of the datasets and the traits being studied. There are several reasons why weighting markers might be beneficial for some datasets and traits, but not for others: (1) For traits controlled by many genes with small effects, weighting markers can help identify and prioritize the most influential markers, enhancing prediction accuracy, whereas traits controlled by a few genes with large effects benefit significantly from weighting these key markers, as they have a disproportionate impact on the trait. However, when a trait is controlled by a very large number of genes with very small effects, the benefit of weighting individual markers diminishes as the effects are too dispersed. (2) When marker density is high, weighting can help manage the large volume of data by emphasizing markers with greater predictive power. (3) Weighting markers in regions with high LD can reduce redundancy and focus on the most informative markers; in populations with clear substructures or relatedness, weighting can help correct for population stratification and relatedness, leading to more accurate predictions.

Furthermore, for traits with significant genotype-by-environment interactions, weighting markers that show consistent effects across environments can improve prediction robustness, and for traits with high heritability, certain markers will have strong associations with the trait, making weighting beneficial to capture this strong genetic signal. When a trait is controlled by a very large number of genes with very small effects, the benefit of weighting individual markers diminishes as the effects are too dispersed.

In summary, weighting markers in genomic prediction can enhance model performance by emphasizing the most informative genetic markers, correcting for population structure, and managing high-dimensional data. However, its effectiveness depends on the genetic architecture of the trait, marker density, population characteristics, and environmental stability. Understanding these factors can guide the decision on whether to apply marker weighting in genomic prediction studies.

### 4.2. Specific Considerations for Genomic Prediction

In the context of genomic prediction, predicting traits based on genetic markers is complex and often challenging. For example, an NRMSE of 0.015 (see Table 2 and Table A1) is particularly impressive. It indicates that the model predictions are very close to the true values, with only a 1.5% error, that is, with a very small deviation from its target. The larger and more complex the dataset, the more impressive a low NRMSE becomes. Highly variable traits typically result in higher errors, so a low NRMSE here is significant. Simpler models achieving low NRMSEs suggest a robust underlying pattern, whereas complex models might fit noise if overfitting. For example, in a genomic prediction study for plant breeding where breeders are predicting crop yield or resistance to diseases, an NRMSE of around 0.015 means the model’s predictions are very accurate compared to the variability in the observed data.

## 5. Conclusions

The lack of strong relatedness between training and testing sets is a common issue in the practical implementation of GS, significantly affecting its accuracy. To address this problem, we propose a method aimed at improving accuracy under these conditions. Our findings indicate that the proposed method enhances accuracy when there is a significant mismatch between training and testing sets. However, while the method provides improvement, it does not completely resolve the issue. In addition to addressing this issue, we emphasize the significance of the problem within the GS methodology, as it often compromises its efficiency. We encourage other scientists to enhance the proposed framework to further increase the robustness of the GS methodology.

## Figures and Tables

**Table 1:** Description of the datasets used to implement the proposed method.

Dataset Number	Dataset (Crop and Number)	Cultivars (Number)	Markers (Number)	Environments (Number)	Traits (Number)	Families (Number)
Dataset 1	Maize_1	1000	4085	11	4	6
Dataset 2	Maize_2	1000	4085	11	4	6
Dataset 3	Maize_3	1000	4085	11	4	6
Dataset 4	Maize_4	999	4085	11	4	6
Dataset 5	Soybean_1	1044	1810	8	6	10
Dataset 6	Soybean_2	691	1808	8	6	10
Dataset 7	Soybean_3	70	1809	8	6	10
Dataset 8	Soybean_4	59	1803	8	6	10

Data availability: The data are available at: https://github.com/osval78/Mistmach_Penalized_Regression (accessed on 21 July 2024).

**Table 2:** Prediction performance in terms of normalized root mean square error (NRMSE) for datasets Maize_1, Maize_3, Sobybean_1, and Sobybean_2. ENet denotes Elastic net regression, Lasso denotes Lasso regression, and Ridge denotes Ridge regression, while WENet, WLasso, and WRidge denote the weighted counterparts of ENet, Lasso, and Ridge. NRMSE_SD denotes the standard deviation of the NRMSE.

Dataset	Model	Trait	NRMSE	NRMSE_SD
Maize_1	ENet	GDD_ASI	1.427	0.010
Maize_1	ENet	GDD_DTT	1.429	0.010
Maize_1	Lasso	GDD_ASI	1.433	0.011
Maize_1	Lasso	GDD_DTT	1.433	0.010
Maize_1	Ridge	GDD_ASI	1.371	0.009
Maize_1	Ridge	GDD_DTT	1.371	0.009
Maize_1	WENet	GDD_ASI	1.307	0.005
Maize_1	WENet	GDD_DTT	1.307	0.005
Maize_1	WLasso	GDD_ASI	1.332	0.006
Maize_1	WLasso	GDD_DTT	1.332	0.006
Maize_1	WRidge	GDD_ASI	1.303	0.006
Maize_1	WRidge	GDD_DTT	1.303	0.006
Maize_3	ENet	GDD_ASI	1.405	0.011
Maize_3	ENet	GDD_DTT	1.404	0.011
Maize_3	Lasso	GDD_ASI	1.409	0.011
Maize_3	Lasso	GDD_DTT	1.407	0.011
Maize_3	Ridge	GDD_ASI	1.380	0.010
Maize_3	Ridge	GDD_DTT	1.381	0.010
Maize_3	WENet	GDD_ASI	1.369	0.007
Maize_3	WENet	GDD_DTT	1.369	0.007
Maize_3	WLasso	GDD_ASI	1.370	0.008
Maize_3	WLasso	GDD_DTT	1.370	0.008
Maize_3	WRidge	GDD_ASI	1.331	0.008
Maize_3	WRidge	GDD_DTT	1.333	0.008
Soybean_1	ENet	R8	1.241	0.003
Soybean_1	ENet	Height	1.193	0.003
Soybean_1	Lasso	R8	1.254	0.003
Soybean_1	Lasso	Height	1.195	0.003
Soybean_1	Ridge	R8	1.270	0.004
Soybean_1	Ridge	Height	1.143	0.002
Soybean_1	WENet	R8	1.274	0.003
Soybean_1	WENet	Height	1.239	0.003
Soybean_1	WLasso	R8	1.255	0.003
Soybean_1	WLasso	Height	1.227	0.003
Soybean_1	WRidge	R8	1.284	0.004
Soybean_1	WRidge	Height	1.191	0.003
Soybean_2	ENet	R8	1.554	0.004
Soybean_2	ENet	Height	1.361	0.002
Soybean_2	Lasso	R8	1.559	0.004
Soybean_2	Lasso	Height	1.357	0.002
Soybean_2	Ridge	R8	1.545	0.004
Soybean_2	Ridge	Height	1.342	0.002
Soybean_2	WENet	R8	1.470	0.003
Soybean_2	WENet	Height	1.316	0.002
Soybean_2	WLasso	R8	1.473	0.003
Soybean_2	WLasso	Height	1.313	0.002
Soybean_2	WRidge	R8	1.515	0.004
Soybean_2	WRidge	Height	1.332	0.002

**Table 3:** Prediction performance across all traits in terms of normalized root mean square error (NRMSE) across the eight datasets. ENet denotes Elastic net regression, Lasso denotes Lasso regression, and Ridge denotes Ridge regression, while WENet, WLasso, and WRidge denote the weighted counterparts of ENet, Lasso, and Ridge. NRMSE_SD denotes the standard deviation of the NRMSE.

Model	NRMSE_Mean	NRMSE_Mean_SD
Enet	1.409	0.001
Lasso	1.413	0.001
Ridge	1.387	0.001
WENet	1.378	0.001
WLasso	1.377	0.001
WRidge	1.369	0.001

## Data Availability

The original data presented in the study are openly available at https://github.com/osval78/Mistmach_Penalized_Regression (accessed on 21 July 2024).

## Appendix A

**Table A1:** Prediction performance in terms of normalized root mean square error (NRMSE) for datasets Maize_2, Maize_4, Sobybean_3, and Sobybean_4. ENet denotes Elastic net regression, Lasso denotes Lasso regression, and Ridge denotes Ridge regression, while WENet, WLasso, and WRidge denote the weighted counterparts of ENet, Lasso, and Ridge. NRMSE_SD denotes the standard deviation of the NRMSE.

Dataset	Model	Trait	NRMSE	NRMSE_SD
Maize_2	ENet	GDD_ASI	1.669	0.012
Maize_2	ENet	GDD_DTT	1.669	0.012
Maize_2	Lasso	GDD_ASI	1.674	0.012
Maize_2	Lasso	GDD_DTT	1.674	0.012
Maize_2	Ridge	GDD_ASI	1.647	0.013
Maize_2	Ridge	GDD_DTT	1.647	0.013
Maize_2	WENet	GDD_ASI	1.662	0.013
Maize_2	WENet	GDD_DTT	1.662	0.013
Maize_2	WLasso	GDD_ASI	1.679	0.013
Maize_2	WLasso	GDD_DTT	1.679	0.013
Maize_2	WRidge	GDD_ASI	1.571	0.012
Maize_2	WRidge	GDD_DTT	1.571	0.012
Maize_4	ENet	GDD_ASI	1.544	0.011
Maize_4	ENet	GDD_DTT	1.546	0.011
Maize_4	Lasso	GDD_ASI	1.550	0.011
Maize_4	Lasso	GDD_DTT	1.547	0.011
Maize_4	Ridge	GDD_ASI	1.544	0.012
Maize_4	Ridge	GDD_DTT	1.546	0.012
Maize_4	WENet	GDD_ASI	1.501	0.010
Maize_4	WENet	GDD_DTT	1.502	0.010
Maize_4	WLasso	GDD_ASI	1.513	0.011
Maize_4	WLasso	GDD_DTT	1.513	0.011
Maize_4	WRidge	GDD_ASI	1.465	0.011
Maize_4	WRidge	GDD_DTT	1.464	0.011
Soybean_3	ENet	R8	1.250	0.028
Soybean_3	ENet	Height	1.104	0.009
Soybean_3	Lasso	R8	1.276	0.030
Soybean_3	Lasso	Height	1.097	0.009
Soybean_3	Ridge	R8	1.264	0.034
Soybean_3	Ridge	Height	1.093	0.009
Soybean_3	WENet	R8	1.278	0.033
Soybean_3	WENet	Height	1.097	0.015
Soybean_3	WLasso	R8	1.264	0.032
Soybean_3	WLasso	Height	1.069	0.009
Soybean_3	WRidge	R8	1.292	0.037
Soybean_3	WRidge	Height	1.085	0.011
Soybean_4	ENet	R8	1.090	0.010
Soybean_4	ENet	Height	1.176	0.019
Soybean_4	Lasso	R8	1.146	0.010
Soybean_4	Lasso	Height	1.180	0.020
Soybean_4	Ridge	R8	1.066	0.008
Soybean_4	Ridge	Height	1.121	0.018
Soybean_4	WENet	R8	1.127	0.010
Soybean_4	WENet	Height	1.156	0.013
Soybean_4	WLasso	R8	1.116	0.010
Soybean_4	WLasso	Height	1.132	0.013
Soybean_4	WRidge	R8	1.069	0.008
Soybean_4	WRidge	Height	1.129	0.015
